# Transnational migrants' philanthropy: Its forms, operations, and implications from the perspectives of Ghanaian residents in Europe

**DOI:** 10.3389/fsoc.2022.1062755

**Published:** 2023-01-18

**Authors:** Mercy DeSouza, Onallia Esther Osei, Erhabor Sunday Idemudia

**Affiliations:** ^1^Faculty of Management Studies, University of Professional Studies, Accra, Ghana; ^2^Faculty of Arts and Social Sciences, Maastricht University, Maastricht, Netherlands; ^3^Faculty of Humanities, North-West University, Mmabatho, South Africa

**Keywords:** giving back, Ghana, transnational migrant philanthropy, remittances, hometown associations, religious or faith-based associations

## Abstract

**Introduction:**

With the emergence of transnational migration studies in the 1990's, migration studies became involved in showing how migrants maintain transnational connections through money and non-monetary philanthropic contributions in their origin countries. However, there is little evidence about the interconnections between different forms of migrants' philanthropy and how they are developed and sustained over time across international borders.

**Methods:**

This work investigates individual and groups transnational philanthropy and shows how migrants become involved in these forms of philanthropy, highlighting some changes therein over time. We relied on fifty semistructured interviews and six focus group discussions conducted with Ghanaians in the Netherlands, Italy and Germany.

**Results and discussion:**

Our thematic analyses confirm that transnational migrant philanthropy is about fulfilling certain “moral obligations,” to derive a sense of belonging “here” (destinations) and “there” (origins). In performing the self, religious or culturally imposed sense of responsibility for human welfare and institutional development in the home country, Ghana, involved migrants overcome some challenges. For transnational migrant philanthropy to sustain itself, studied migrants think origin country governments must take necessary steps to remove structural obstacles like tedious procedures for clearing philanthropic goods at the ports and harbors. Involved migrants also suggested a need for a more organized platform to collect relevant information on potential beneficiary needs for their preparations to “give back” to their homeland.

## 1. Introduction

When faced with life challenge like poverty (economic hardships), political persecution and adverse environmental factors like inadequate water supply and degraded farmland, modern Ghanaians sometimes embark on an international search for better living conditions and economic opportunities for themselves and their families. During international migration, Ghanaians support others back “home” through monetary remittances where possible or desirable (Awumbila et al., 2011). Though not in comparative terms as the attention given to migrant remittances and development in origin countries, migrant philanthropy has recently gained much attention in academic and policy settings. This paper contributes to the limited knowledge about migrant philanthropy dynamism.

Migrant philanthropy enables some migrants to build and sustain their transnational identities to their own or family birthplace (Lacroix, 2016; Akom-Ankobrey et al., 2022). Migrant philanthropy consists of all benevolent acts such as charitable donations, gifting, giving back and volunteering for a common good (Schuyt, 2013, p. 26; Drouhot et al., 2022). The main driving forces for migrant philanthropy are giving back to remain connected to their or family's origin countries, or to fulfill moral, ethical or religious obligations (Ang, 2001; Drzewiecka and Halualani, 2002; Werbner, 2002; Baig, 2016). Despite its development implications, there is little information about how migrant philanthropy sustains itself across time and space depending on involved actors. The scarce literature also discusses individuals and group philanthropy in isolated fashions without systematically showing differences and similarities between these established forms of migrant philanthropy as we do in this paper.

Migrants choose to donate for perceived good courses in their origin countries as individuals or with others as a group in the form of hometown associations or religious or faith-based organizations (Ogden and Mazzucato, 2021). Migrants' financial remittances influence economic development in their origin countries (e.g., Asare, 2012; Baig, 2016; Espinosa, 2016; Kumi, 2019). Mobile (visiting and returning) migrants sometimes take up expert positions in origin countries or homelands to provide their quota to such nation-state's development (Simoni and Voirol, 2021). Other migrants remit to their family members for basic needs, family projects, or to previous social networks in homelands as private social welfare provision (e.g., Ademolu, 2021). As many publications have focused on migrants' motivations to employ philanthropy to establish a transnational sense of belonging, there seems to be an oversight on other issues involving their experiences with various forms of philanthropy and the implication therein.

Therefore, this research moves existing knowledge about migrants' philanthropy forward by asking these overarching questions: what are the operational issues therein individual or group of migrants' philanthropy, and how do involved migrants try to address obstacles or impediments related to their philanthropic engagements with their origin country? What are the successful occurrences of activities performed to overcome encountered hurdles? Based on encountered success or shortcomings, how do migrants want their origin countries to be set up for smooth implementations of their philanthropic plans and activities as grounds for developing and sustaining their transnational ties between destination and origin countries? By doing so, we respond to migration studies advancement calls to distinguish between different types of transnational activities with the migrants' agency at the center of analysis and discussion (van Meeteren, 2012). To fill these identified lacunae in knowledge, we show how individual and group transnational migrant philanthropy relates and departs from each other based on these structural findings (motivations, engagements and challenges).

To extend the current understanding of transnational migrant philanthropy, we first provide background literature. Secondly, we discuss our methodology followed by a thorough discussion of our major findings. We conclude by sharing a summary of findings tied with highlighted recommendations.

### 1.1. Applying a transnationalism perspective to the migrant philanthropy debate

For about a century, transnationalism has predominated in Ghanaians' international migration studies (Awumbila et al., 2011), but scarcely about Ghanaian migrants' philanthropic activities. As a result, this article also applies the concept of transnationalism to show Ghanaian migrants' connections across national boundaries through “giving back” activities (e.g., Akom-Ankobrey et al., 2022). A transnational lens helps differentiate “below” social relations, movements, and realities based on migrants' perspectives from “above” globalization by corporations or states (Guarnizo and Smith, 1998). Transnationalism applied to migration studies refers to how migrants forge and sustain multi-stranded relations linking origin and receiving countries across geographical boundaries through socio-cultural, commercial and political linking activities (Basch et al., 1994, p. 6). For example, Ghanaians abroad are well noted for sharing their economic wealth with family members by sending remittances and hometown micro-development projects like boreholes (e.g., Mazzucato, 2008; Awumbila et al., 2011; UNDP, 2017; Kumi, 2019). Some Ghanaians abroad also “give back” through skills training and capacity building, water and sanitation, health, agriculture and food security, microfinance, education, and representation in policy process (Ong'ayo, 2014).

Currently, migrant philanthropy is a developing field of study mostly applying transnationalism to understand individuals and groups' donations for diverse projects in origin countries (e.g., Escobar, 2015; Santamaria-Alvarez and Sliwa, 2016). Migrant philanthropy is “how” migrants give directly for “charitable purposes,” or family obligations, and “indirect giving to a foundation, public charity or any intermediary for social investments” in origin countries (Lethlean, 2003, p. 14). This article occasionally uses “transnational philanthropy” in place of migrant philanthropy to refer to migrants' participation in development related activities through remittances and other non-monetary transnational contributions toward their origin countries.

The transnational flow of migrant philanthropy guarantees neoliberal economic, social, and cultural ideologies about people's moral duties to promote social welfare as development agents for their homeland or native countries (e.g., Tchouassi and Sikod, 2010; Pharoah et al., 2013; Baig, 2016; Nwadiuko et al., 2016; Keles, 2022). In line with this point of view, we rely on remittance literature for a richer comprehension about morality and family relationships, personal accountability and development. Such a viewpoint offered us an opportunity to look at ethical and value systems shaping migrants' realities to try and give back to their origin countries. By doing so, we could develop moral arguments about being a transmigrant philanthropist, differences in migrants' values, experienced ambivalences, and other challenges. For some migrants' (Africans, Asians and Latinos) “giving back” is a way to honor, respect, and express thanks to one's parents and grandparents, preserve the reputation of one's family, or ensure the socioeconomic wellbeing of one's family and community (Ademolu, 2021). Some migrants from the three stated origin blocks practice transnational philanthropy through their religious or faith-based organizations, ethnic and professional groups, hometown associations, and internet giving platforms (e.g., Simoni and Voirol, 2021; Mutambasere, 2022). In the later body of literature, home-town associations are the subject of most research (e.g., Orozco, 2003; Orozco and Lapointe, 2004; Merz, 2005; Mazzucato, 2008; Escala-Rabadán et al., 2011).

Overall, migrants' reasons to contribute to development related activities in their origin countries are to fulfill moral, ethical or religious commitments (Werbner, 2002), for real or symbolic transnational ties (Ang, 2001, p. 25; Baig, 2016). Such a transnational implication encapsulates acquiring “a sense of belonging and connection” to origin countries or hometowns (Drzewiecka and Halualani, 2002, p. 340–341). The existing literature mostly centers on migrants' motivations to engage in specific philanthropy toward their origin countries, however, not much has been done through comparative design to enrich academic understanding as there are calls to do (e.g., Santamaria-Alvarez and Sliwa, 2016). In this article, we investigate both individual and group transnational migrant philanthropies to highlight distinctions and affinities within and between the observed classifications.

Transnational migrant philanthropy involves contributing relatively small to huge sums of money for diverse purposes and projects in their origin countries (Flanigan, 2017; Brinkerhoff et al., 2019). There are also material donations costing little or much when converting into monetary values, and non-material aid (e.g., Simoni and Voirol, 2021). It is also vital to acknowledge facilitation roles of technological tools like mobile phones and internet for gathering the appropriate information to formulate philanthropic ideas, execution plans and sometimes used for parts of the actual execution, including technologies usefulness for migrants' engagements and money transfers to families, neighbors, and friends (e.g., Baig, 2016). To carry out development projects in their home countries, some transnational migrants engage in philanthropy by organizing fundraising events in the receiving or destination nations (Flanigan, 2017; Babis et al., 2021). Governments of origin countries encourage their migrant and diaspora communities to be enthusiastic about “individual remittance” and “collective remittance” or “organized association remittances” comprising of giving back as faith-based organizations, hometown associations and as immigrant friends or neighbors (Lacroix, 2016; Mekonnen, 2018; Chimienti and Solomos, 2020).

We advance existing knowledge about transnational migrant philanthropism by centering migrants' agency for transnational practices (van Meeteren, 2012; Crawford and Martin, 2014; Kamaras, 2022), thereby showing how different migrants irrespective of their socio-economic backgrounds in destinations countries opt to become transnational philanthropist. By investigating the interconnections between studied migrants' philanthropic engagement motivations, challenges and coping strategies over time, we highlight some shifts from individual to group philanthropy. By doing so, we show the diversity of migrants' initiatives and sustainable practices related to transnational philanthropy toward origin countries. We assume that through continuous giving back or philanthropy, migrants develop more passion, enthusiasm, zeal and dedication about being involved in, committed to and absorbed by developmental goals in their origin countries while they reside abroad or overseas for varied reasons. Some migrants who seem ephemeral incapable based on their destination socio-economic conditions might choose to find solace through migrant philanthropy to cope with the difficulties of residing abroad.

## 2. Methodology

This article extends prevailing knowledge on transnational migrants' philanthropy using qualitative data from three European field sites: The Netherlands (Amsterdam), Germany (Berlin) and Italy (Rome). We selected these countries due to their proximity within the European Union to ease data collection. These countries have also been cited as major migration destinations for Africans [International Organization of Migration (IOM), 2017]. There is abundant evidence that Ghanaian migrants in the three studied European countries maintain ties to their homeland through remittances and travel (Idemudia and Boehnke, 2020; Ogden and Mazzucato, 2021; Akom-Ankobrey et al., 2022). We chose Ghana for this research because of the absence of a trusted framework on transnational migrant philanthropy in Ghana and limited knowledge on their philanthropic activities (Kumi, 2019). As Ghanaians, we could also leverage our proficiency in the Ghanaian languages to strengthen our data collection as well as our social networks to facilitate field access.

The study was guided by the interpretivist paradigm, aimed at eliciting and understanding migrants' experiences of philanthropy, therefore the qualitative design was deemed most appropriate. We conducted 50 individual interviews and 6 focus group discussions (FGDs) with a total 54 focus group participants (hometown and religious groups) in June – September 2019. These included 21 individual interviews in Berlin, 10 in Amsterdam and 19 in Rome and 2 focus group sessions in each city.

The researchers worked with Ghanaian residents in Amsterdam, Berlin and Rome to recruit participants and assist with data collection. For inclusion, an interested participant must identify themselves as first generation migrant irrespective of citizenship, must have continuously lived in the host country for at least 1 year, aged 18 years or older. To give different Ghanaian ethnicities a chance to participate beyond languages that authors spoke, we decided to allow any interested participants who could speak English in any style, including pidgin, a chance to participate. We also encouraged participants to use Ghanaian words whenever they felt it was needed or it was the best way to express themselves about an issue being discussed in the individual interviews and focus group discussions.

With the inclusion criteria in mind, we relied on purposive and snowball sampling for participants' recruitment. Participants were approached at African shops, Ghanaian churches, hometown group meetings, the Ghanaian embassies and university campuses. Once someone qualified by the selection criteria, they received consent information to affirm or disaffirm their participation. Interview times and locations were thereafter agreed upon with interviewees. Participants' length of stay in Europe ranged between 1 and 31 years. While some of the participants were graduate workers living abroad, others had no to little education based on the European standard. The latter often performed menial jobs which offer unstable employment. Some participants preoccupied themselves with multiple jobs to make ends meet while others were unemployed due to unemployment or old age.

Once data collection ended, we transcribed the audio recordings for coding and thematic analysis. In line with Braun and Clarke's (2006) thematic analysis approach, we read through and transcribed documents for coding aided by the MAXQDA 2020 software. For anonymity and confidentiality, identifiers denoting country of interview, participants gender and a unique number were used for each individual transcript. For example, an individual transcript coded as “GM7” means the interview was conduct in Germany (G) and the interviewee a male (M) was the 7^th^ participant. Guided by the research questions and literature reviewed, phrases, words and paragraphs were assigned codes. Such coding also provided us chances to gain a general understanding of migrant's experiences. Through constant comparison, codes were collapsed and merged into themes for the desirable analysis by the first two authors. Discussions between these two researchers resulted in many interactions with the coded data until we arrived at the shared findings through further deliberations based on the data among all three authors.

## 3. Findings and discussions

We identified the following overarching themes for discussion: (1) Migrants motivation for philanthropy (individual, hometown and religious groups' motivation); (2) nature of philanthropic engagements; and (3) observed challenges and recommendations. The rest of this section outlines and explains identified themes with a selection of pseudonymised participant quotations.

### 3.1. Migrants motivation for philanthropy

#### 3.1.1. Individual philanthropists' motivation

The majority of the individual philanthropists in our study reported that (1) desiring to give to the needy, (2) feeling obliged to give to avoid social exclusion, and (3) giving out of empathy based on their past experiences motivate them to give back to people and institutions in their origin country. Thus, while some individual philanthropists gave out of sentimental or emotional reasons; others give to impress family, thereby earning the required respect to maintain family ties. A male migrant, who has lived in the Netherlands for 19 years articulated that: *Nowadays, aborokyiri (Europe) is not what it used to be. My nieces and nephews are even richer than myself. Working as tillers and Masons, some have built. But I provide for them because it is a must do. When you stop sending to them, they will call you and remind you “kai fie (remember home) and even warn you that if you stop caring, they will stop considering you as a part of the family”* (Interview NM9). These results confirm migrants giving back to maintain a sense of feeling connected to the home country, thereby avoiding potential social sanctions (Simoni and Voirol, 2021; Akom-Ankobrey et al., 2022; Drouhot et al., 2022).

In another case, a female migrant in Berlin who migrated for family reunion narrated her family's ordeal in caring for their autistic child in the origin country before traveling abroad. With such an emotional experience constantly on this migrant family's mind, they willingly give to support the Dzorwulu special needs school in Ghana. This was echoed in another way by a male migrant in Rome whose motivation to give is based on an experience of lack of medical facilities that resulted in a relative's death in Ghana: “*I contribute to the Ghana heart foundation, my elder brother died in Ghana from a heart condition and since then, 5 years until now, I have been contributing”* (Interview IM16). Although these observations resonate well with sentimental giving, they represent how migrants turn personal or family sentiments into transnational migrant philanthropy by an individual or a group of people, herein, not as a hometown or religious body. This situation does not necessarily mean that the other philanthropic groups do not embark on sentimental transnational philanthropy.

#### 3.1.2. Hometown group's motivation

Hometown group philanthropy was driven by (1) the desire to meet necessary community needs, (2) to maintain ties with the hometown for mutual benefit, and (3) the inclination to protect themselves from spiritual (evil) attacks, which they believe is imminent in individual philanthropy. Though the fulfillment of meeting hometown needs and maintaining ties were identified as key motivators for HTGs engagement in philanthropy, migrants also indicated that receiving physical benefits and recognition from beneficiaries intensifies their desire to help the community. For example, one group's member in Rome confirmed that: “*we got plenty plots of land from a chief after we helped the community to get a school and a borehole. So, we also get some good from doing good. When you know the benefits, you want to do more”* (Interview HTG1, Rome). This observation supports the assertion that migrants cluster in destination countries as hometown groups to mimic patterns of supportive social relationships they experienced in origin communities or expect from their ethnic, clan or tribe members (Lamba-Nieves, 2018; Babis et al., 2021; Kamaras, 2022). So, giving back to the hometown which is a sort of a tribal, ethnic or clan origin relates to an expectation to receive from clan, ethnic or tribe members in the destination country.

Group philanthropy was also mentioned as a buffer against jealousy and the “evil eye” (the tendency for someone to bewitch) as it erases some personal recognition and association linked to individual philanthropy. As explained by a hometown group in Amsterdam: “*We belong together, and we feel good if we can come together to give to our motherland. Trying to do it alone is like hanging yourself. Evil eyes (Enibone) will come on you in Ghana. They will drag you back. When we do it together it prevents jealousy”* (HTG2 Amsterdam). Perceptions like these weaken migrants' desires to engage in individual philanthropy as voiced by another migrant: “*Apart from my small monies [sent to] my immediate family, I cannot do anything ankoman (alone). If I attempt, they will think am very rich even my family members will insult me. I sent some computers to a school in…and my mother actually said I am announcing my wealth. I should be careful”* (HTG2 Amsterdam) Those who transitioned from individual philanthropy to group philanthropy were strongly of the view that the people in their origin countries will witch hunt them if they continue to voluntarily give back as sole philanthropists and implement communities' projects as the home town associations do.

#### 3.1.3. Religious group's motivation

Religious groups indicated (1) advancing of religious faith by gaining converts and (2) fulfilling their spiritual obligation to give as the main drivers of their philanthropy. Premised on the Christian religious belief that God blesses a cheerful giver; the religious group migrants stated they felt obliged to sacrifice part of their earnings to take care of needy communities in towns where their “sister” churches are located in Ghana. As remarked by a group participant: “*charity is important, and the bible makes me understand that when we give, we are paid back. So, helping the poor makes me feel like a good and whole Christian”* (RG1, Berlin). This assertation connotes that similar to Ademolu (2021) and Mutambasere (2022) views, religious philanthropy is an exchange between the giver and beneficiaries, where the giver assumes the position of being a channel of distribution of resources to the needy, for egoistic or purely altruistic reasons. Also, to comply with tenets of their Christian faith, migrants are motivated raise funds to undertake philanthropic projects largely linked to getting more converts into their religious faith. This implies that the chunk of migrants' philanthropic contributions may circulate within their own religiously affiliated communities.

#### 3.1.4. Overall findings regarding migrants' motivation for philanthropy

Majority of the migrants studied willingly engage in philanthropy, without cohesion, to increase living standards of beneficiaries although this situation sometimes presents a false impression about migrant philanthropists' socio-economic statuses in the origin country. Some migrants give back based on the peculiarities of their pre-migration challenge which pushes them to feel emotionally connected to the plight of beneficiaries with similar experiences in the origin country. While some individual philanthropists give under coercion to fulfill their moral and social obligation and to avoid family displeasure, religious groups similarly give to fulfill their spiritual obligation and to avoid displeasing God. An important distinction found was that while group philanthropists were encouraged by the recognition from beneficiaries, conversely, recognition was regarded as detrimental and demotivating for some individuals' philanthropists thus causing them to sway to group philanthropy. Our findings on migrant groups' philanthropic motivation, juxtaposed with the findings on individual philanthropists' motivation' strengthen arguments that migrant philanthropy is an exchange relationship motivated either by purely altruistic or egocentric reasons, and shows some interconnections between individual and group philanthropic motivations.

### 3.2. Nature of migrant philanthropic engagements

Helping relatives and friends in need is the first and foremost type of individual philanthropy. As earlier depicted, this may be because individual philanthropic motivation was said to be particularly influenced by pressure from family and significant others. This distinction in giving behavior can also be explained with the assumption made by Santamaria-Alvarez and Sliwa (2016) that individuals give back more often to close relations because of the relationship with the beneficiary. A few individual philanthropists also targeted or partnered with local foundations or charity organizations, classmate unions and churches. Some charity organizations in our data are orphanages and physically challenged homes, where migrants donated food items, clothing, educational supplies or offered pro bono services such as training in hospice.

Unlike individual philanthropists who gave intermittently due to constraint on their finances, groups relied on collective donations to give strategically and regularly. For example, all the hometown groups interviewed indicated that they had adopted hospitals or clinics where they help with regular renovations and consistently donate both used and new medical equipment and supplies. Hometown groups were also engaged in other community development projects such as renovating or constructing classroom blocks, boreholes' drilling and provision of sanitary facilities to their communities. One hometown group mentioned that with the help of traditional leaders, they also regularly finance community education on health and other social vices. As explained by the leader of the hometown group: “*When Ebola came, we organized students to clean up and inform the town about Ebola. Because of the food and T-shirts, we gave to them, they worked hard. Our group is called… [name of hometown withheld] …. Mansaamo ke hewale Kpee. Our name means we want our town to be clean and healthy.”* Similar to findings of Appe and Oreg (2020) studies, these hometown groups depended on traditional authorities and personal networks for information about community needs and to also coordinate the philanthropic initiatives.

With an overarching goal of promoting Christian faith and converting “unbelievers” to Christ, i.e., promoting salvation related to Christianity, majority of the projects undertaken by religious groups centered on evangelistic missions, medical outreaches, and community development carried out through affiliated religious denominations in the home country. One religious group leader mentioned that in addition to building infrastructure in remote communities which helps to boost the retention of government workers in these communities, they regularly give 10 percent monthly tithing to branches or start-up churches in these communities. He stated: “*We have built a flat for nurses sent to the village, before the project, nurses refused posting to the village. Now we are building another, just single rooms with shared toilet and bath for teachers posted to the community. W*e *use the charity we give to the community to bring spiritual change, sometimes the same way the early missionaries came [to Africa], when we help the community, we can start churches in these communities*.” This narration depicts observations made by other authors that religious transnationalism facilitates diaspora engagement in home community's charity projects. The quote further demonstrates the argument that charitable giving is not purely altruistic because religious groups normally have an ulterior motive of faith conversion tied to their philanthropy (Chimienti and Solomos, 2020; Babis et al., 2021; Mutambasere, 2022).

In addition, two religious groups indicated that apart from drawing from the church resources to fund philanthropy, they sometimes get items such as, used medical equipment and educational materials (computers and tools) from other charitable organizations in the resident country for onward donations to beneficiaries in Ghana. As explained further by a church elder in Berlin, their registration as NGOs in the destination country, facilitates their relationship with other charitable organizations in the resident country who channel donations through them to Ghana: “*We are recognized and registered as an NGO, so it is easier for us to get donations from hospitals and other NGOs to send home. Sometimes we go and ask but there are times they contact us. We have sent used hospital beds to (name of town withheld) twice*. The above statement emphasizes arguments that migrants' philanthropy does not only meet the needs of origin countries and the migrants themselves, but also, promotes migrants' recognition and integration in destination countries (Espinosa, 2016; Brinkerhoff et al., 2019; Chimienti and Solomos, 2020).

Notably, though individual and group philanthropy was geared toward filling in gaps in structural development, health and education of the home country, health related needs was mentioned as the most significant area of concern and focus for most of the migrants. Apart from giving to relatives, Individual philanthropists also give directly to beneficiaries not necessarily linked to their hometowns or religious faith. It was also noted that majority of individuals who chose to be sole philanthropists indicated they also participate in either hometown or religious group philanthropy. As discussed earlier, migrant philanthropy is an exchange relationship, some migrants may participate in all the different forms of philanthropy to derive different forms of satisfaction.

### 3.3. Challenges associated with implementing migrant philanthropy and proposed solutions

Migrant groups fund their giving through collective membership dues, fund raising and church offerings, while individual philanthropists mostly draw on their meager personal finances to solely fund their philanthropic activities. Emotional satisfaction, appreciation and physical benefits derived from philanthropy sustains migrant's motivation to give.

Most of the migrants considered lack of coordination and misuse of their hard-earned income as the main demotivating challenge to philanthropy. Though some religious groups mentioned they consistently transfer cash donations to some churches back home, majority of the hometown groups were strongly opposed to cash donations due to their experiences with financial mismanagement by beneficiaries. Memories of previous philanthropic projects lying waste in the communities and accounts of misappropriation of funds were recalled by a migrant who has lived in Amsterdam for 32 years: “*I like it that when I give to build the classroom or help to repair anything in the community, they name it after me. But it is sad when I go back, and they have not taken care of the building. I have built 4 classrooms so far. When it needs repairs, they wait for me. Even when you send the money, they misuse it.”* This was reiterated by a religious group in Rome: **“***we sent a 20-foot container full of hospital beds and clothe*s. *Most of us do not receive big salaries. After struggling to clear items, I went and found out the things were in the rain because church elders were fighting about where to donate the clothes*”

Studied migrants also lamented that significant others in Ghana are not empathetic to their struggle and hassle in Europe and do not appreciate the sacrifice they make to help the home country. This 44-year-old female migrant who has lived in Berlin for 5 years indicated that she had lost interest in giving due to disappointing experiences. She stated that: “*For me to give to my people back home, I have stopped. They are not grateful if you give and when you even send things home, we pay a lot for the door-to-door service.”* Some migrants mentioned lack of support for their group philanthropic activities from Ghanaian authorities in the destination countries (Ghanaian Embassies) as well as authorities in the origin country, like the Ghana Ports and Harbor authorities. At the time of the interview, one hometown group was in the process of clearing a container of hospital beds they sent to Ghana from the Tema Ports. The leader narrated the frustration they experienced with clearing the hospital beds: “*Though there is a protocol in place at the ports for charities like ours to get a waiver on the things we bring, we have to navigate tedious bureaucratic hurdles, by the time we get the waiver we have huge demurrage charges, it is frustrating*.” Despite the challenges migrants stated they faced in giving back to the home country, most migrants retained the idea of giving back to the home country as a fulfilling practice.

A common recommendation by most migrants was for a proper coordination of their philanthropic activities by the Ghanaian government. For example, migrant of a hometown group in Germany stated that: “*we all put our altekleider (used clothes) in the red cross container. In Ghana they buy these as used clothes on the market. We can put it all together and the government can receive it to send to the children's homes in Ghana. Won't this help a lot?”* In addition, migrants asked for favorable interventions to ensure ease in clearing items and to also ensure that items sent are channeled to the appropriate institution with supervised usage. These are important recommendations because challenges such as misuse of items, high clearing charges and hurdles, misappropriation of community project funds can demotivate philanthropy. In other words, steps must be taken, or interventions must be put in place to remove the demotivating factors to make it more appealing and easier for migrants to engage in giving back to Ghana. The broader implication of these demotivating factors is that Ghana may be losing critical help needed from Ghanaian migrants in Europe to complement government efforts in social, cultural, and economic development.

## 4. Limitations of the study

Our investigation concentrated on three cities in Italy, Germany and the Netherlands, excluding the perceptions of migrants in other European countries. Therefore, the findings in this article may not be generalizable to Ghanaian migrants in other Europeans countries. Some of our studied migrants may not be legally resident, in order not to endanger them, we refrain from publishing the names of hometown and religious groups interviewed. Additionally, the study concentrated on Christian religious groups as most of them had their own places of worship and thus were easily accessible. The views of other Ghanaian religious groups were therefore not represented. Despite these unavoidable limitations, our paper offers an analytical compilation of scholarly ideas about migrants' experiences and opinions on transnational philanthropy. We further detailed motivations for migrants' philanthropic engagements, challenges faced, and how they surmount these challenges to sustain giving as either individual philanthropist or as group philanthropists.

## 5. Conclusion

This paper responds to calls within international migration studies to compare cases concerning migrant philanthropy. Using multiple methods for data collection regarding individual and group philanthropy, we show that migrants who chose to give back as individuals, solely funding their philanthropic acts, equally joined in hometown and religious group philanthropy for varied reasons. Individual philanthropists generally give under cohesion to direct family relations but also engaged willingly in particular philanthropic acts based on peculiar pre-migration challenges. Interestingly, some migrants reported a preference for participation in group philanthropy to forestall perceptions of envy and bewitchment believed to be associated with engaging in individual philanthropy. Our findings validate earlier scholarship indicating interconnections between individual and group philanthropist motivations and tends to agree with studies that reported that though migrants earn scanty salaries in the resident countries and struggle to live comfortably in Europe, yet most migrants aspire to become transnational philanthropist toward their home country to obtain psychological, social and religious benefits from their giving back practices (Sinatti and Horst, 2015; Nwadiuko et al., 2016; Weng and Lee, 2016; Babis et al., 2021). Studied migrants give back to countries of origin with the hope that their giving will address pressing and yet neglected community issues. Sometimes, migrants' donations to individuals and communities within their origin countries does not yield desirable results. Some migrants faced state blockage at Ghana ports and harbors for desirable local impacts through transnational flow of goods like hospital beds. Therefore, the Ghanaian migrants we studied in The Netherlands, Germany and Italy encourage their origin country authorities such as embassies, consulates, ports and harbor authorities to support them actively to engage in giving back to the home country.

Based on the findings, we propose a governance framework on transnational migrants' non-financial contributions to national development in Ghana as this seems to be lacking. Even though non-monetary transnational philanthropy (skills and knowledge transfer likewise social remittance) seems difficult to economically and socially quantify, they contribute to individual and community development in origin countries. The difficulty in quantifying may make it difficult for governments in origin countries to recognize and appreciate the impact of transnational migrants' philanthropy. Putting in place structures to harness, facilitate and ensure the proper usage of migrants' philanthropic contributions will motivate Ghanaian migrants to more willingly contribute their quota to development in their origin countries. Since migrants rely on information about community needs to decide on the direction of philanthropy, it is recommended that origin countries have a reliable database with offices, where allocated officers liaise with migrants to encourage transnational migrant philanthropy.

## Data availability statement

The original contributions presented in the study are included in the article/supplementary material, further inquiries can be directed to the corresponding author.

## Ethics statement

The studies involving human participants were reviewed and approved by the Institutional Review Board at the University of Professional Studies, Ghana with Ethics No: ECUPSA-FM 040-19. Written informed consent for participation was not required for this study in accordance with the national legislation and the institutional requirements.

## Author contributions

MD performed data collection, data analysis, interpretation of results, and manuscript preparation. OO performed data collection, data analysis, literature search, and manuscript preparation. EI facilitated study design, data analysis, interpretation of results, and manuscript preparation. All authors have read and approved final manuscript.
